# Hernia of Morgagni in the Elderly: A Case Report

**DOI:** 10.7759/cureus.1549

**Published:** 2017-08-08

**Authors:** Tushar Patial, Sunil Negi, Vikrant Thakur

**Affiliations:** 1 General Surgery, Indira Gandhi Medical College, Shimla

**Keywords:** morgagni, hernia, diaphragmatic, repair

## Abstract

Congenital diaphragmatic hernias are infrequently encountered in adult patients. A rare type of this hernia is the Morgagni’s hernia. Although they remain asymptomatic in a majority of patients, we present the case of an elderly patient who presented to us with abdominal pain and upper gastrointestinal bleeding.

## Introduction

**Figure 1:**
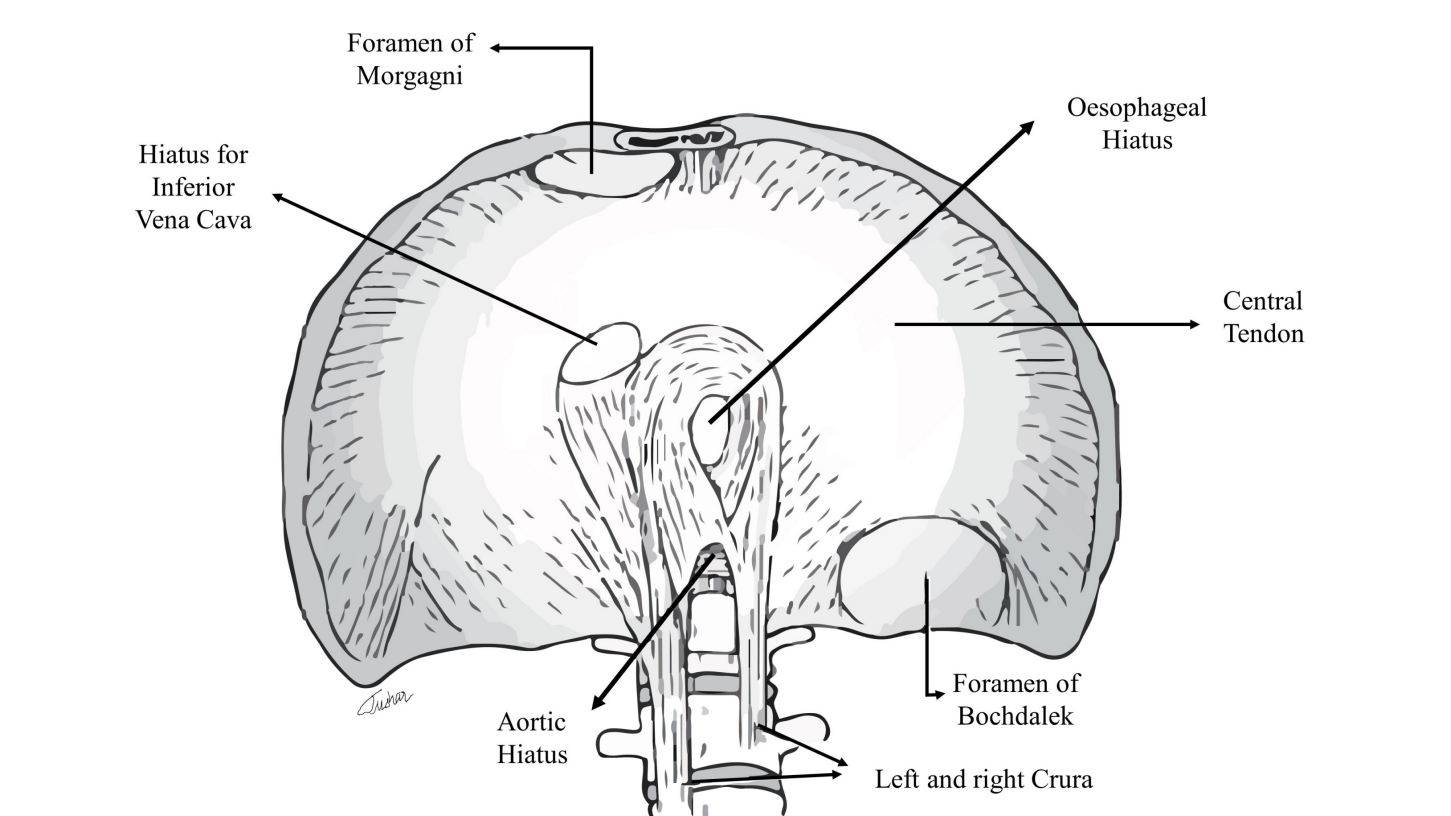
Anatomy of the diaphragm (original illustration)

The foramen of Morgagni is a triangular space located between the muscle fibres from the xiphisternum and the fibres from the costal margin that insert onto the central tendon [1] (Figure 1).

Protrusion of any viscus through this anterior foramen is known as the Morgagni’s hernia (MH). MH is considered to be a paediatric condition since it is congenital in nature. However, there have been many case reports and small series of MH involving adults [2]. Morgagni’s hernia is a rare clinical entity with protean manifestations. Herein, we describe the case of an elderly gentleman who presented to us with this condition.

## Case presentation

**Figure 2:**
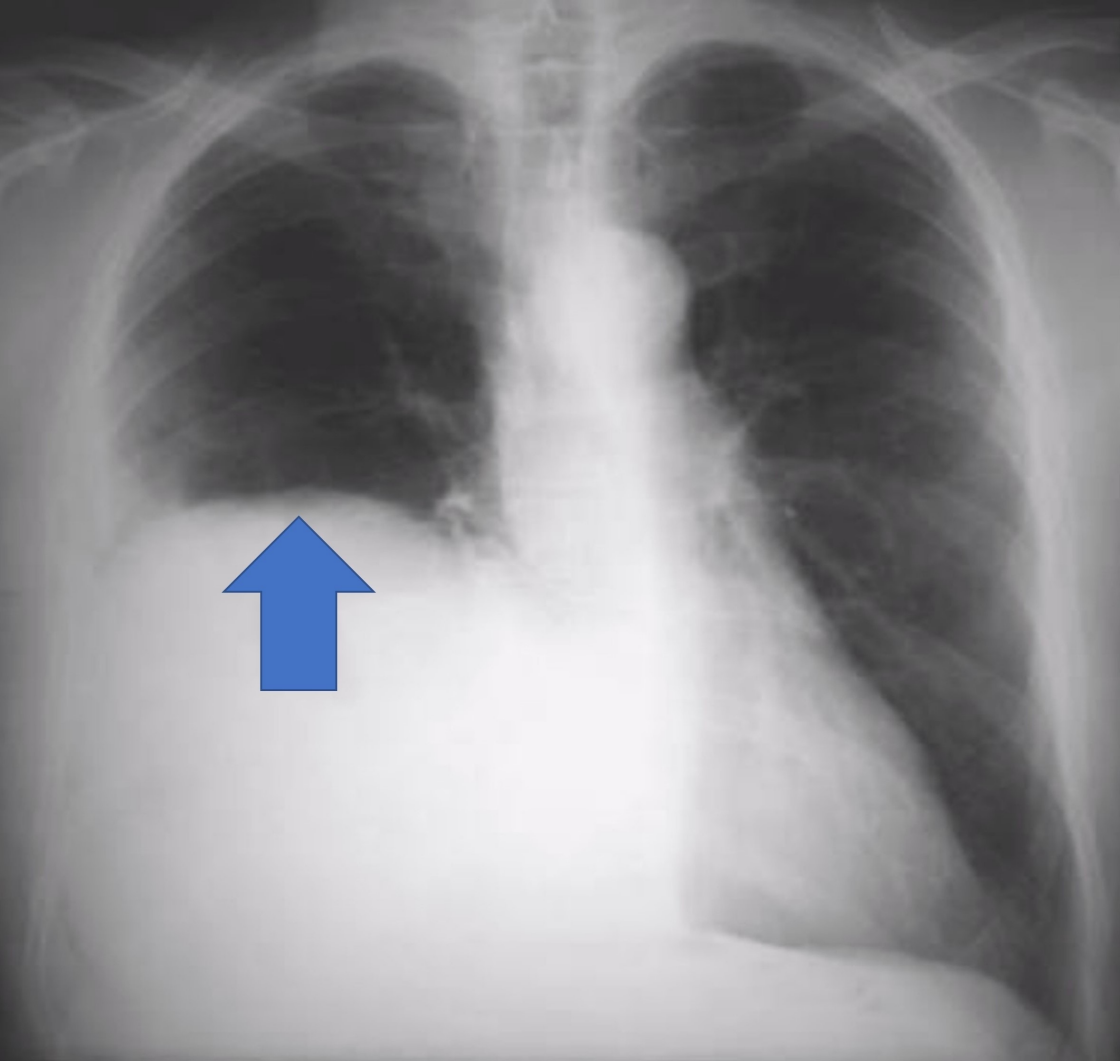
Chest radiograph posteroanterior view showing right dome of diaphragm being raised, with a non-homogenous opacity rising towards the right axilla (blue arrow).

An 82-year-old man presented to the emergency with the chief complaints of progressively worsening upper abdominal pain for three days and multiple episodes of coffee-coloured vomiting for the past eight hours. The pain was constant in nature and no previous episodes were reported. He had 3-4 bouts of coffee-coloured vomiting, but bowel habits were otherwise normal. He gave no history of blood in stools or loose stools. There was no significant past medical history, nor did he have any recent trauma or surgery. On physical examination, the patient had tachycardia and hypotension. Reduced air entry was noted in the right lung base along with the presence of bowel sounds. On per abdomen examination, there was tenderness and guarding over the epigastric region, but no palpable lumps were found. The rectal examination was within normal limits. Blood investigations revealed a total leucocyte count of 14,500 and haemoglobin of 12% with a haematocrit of 40%. Liver enzymes and renal function tests were within normal limits. An arterial blood gas test revealed metabolic acidosis with serum lactate levels of 3.2 mmol/L. Other haematological investigations were normal. A posteroanterior chest radiograph showed the right dome of the diaphragm as being raised, with a non-homogenous opacity rising towards the right axilla (Figure 2).

**Figure 3:**
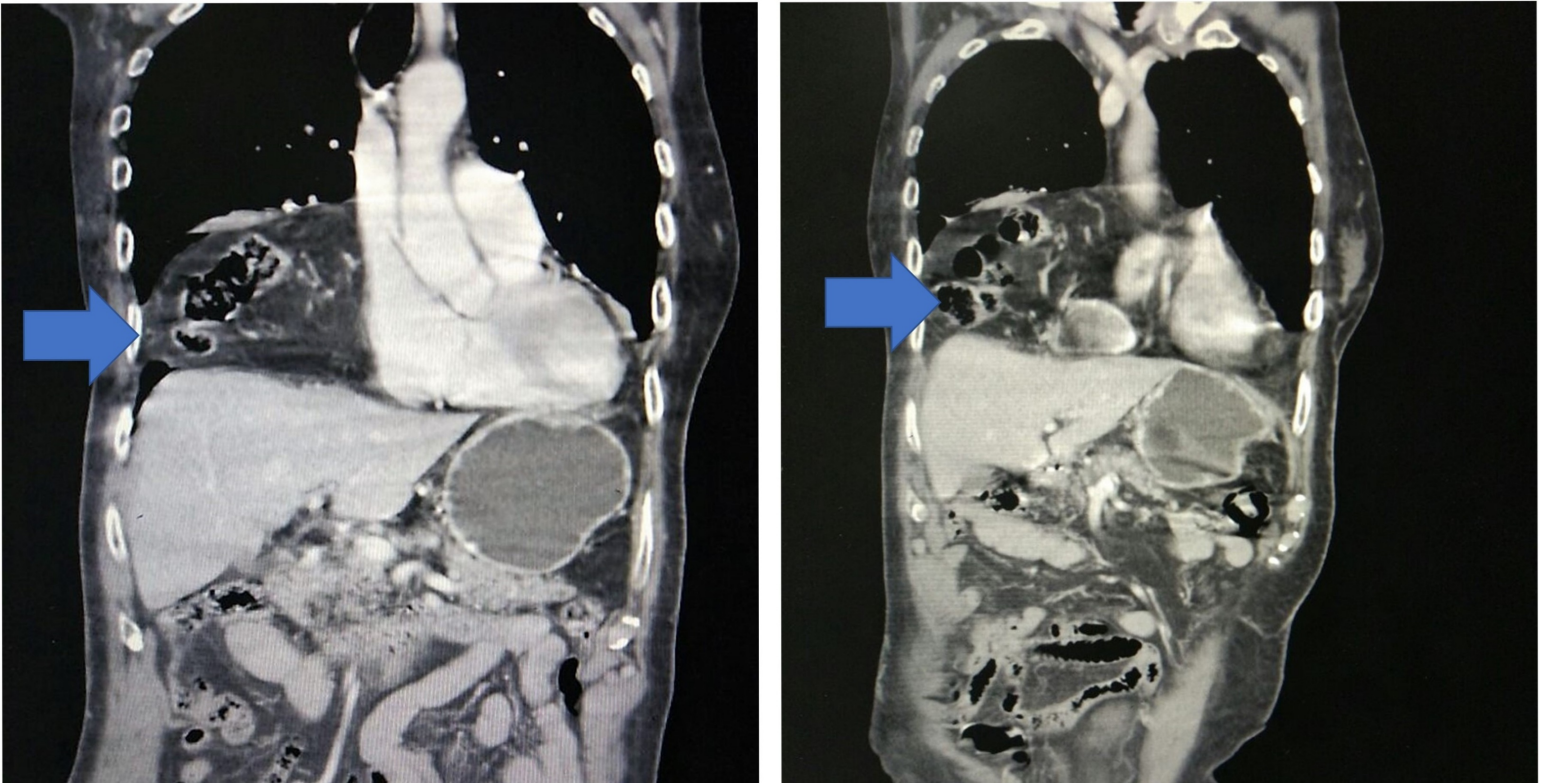
Computed tomography of the chest and the abdomen with diaphragmatic herniation of abdominal contents into the thoracic cavity

Computed tomography (CT) of the chest and the abdomen was performed, with findings suggestive of diaphragmatic herniation of the omentum, pylorus, colon, and suspected duodenum into the thoracic cavity. The gastroesophageal junction was noted to be normal (Figure 3).

**Figure 4:**
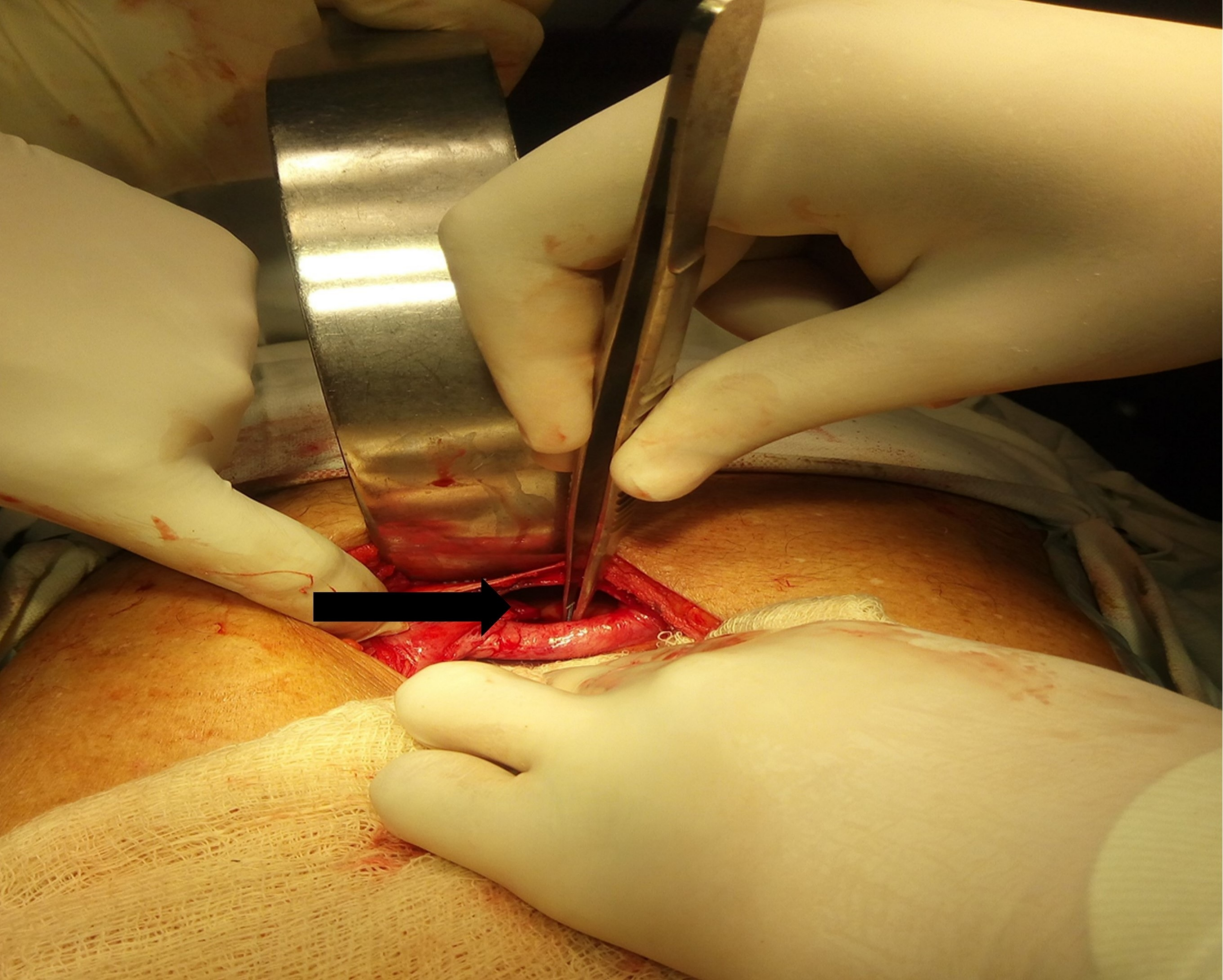
Intraoperative photograph showing central defect just behind xiphisternum (black arrow).

Due to his worsening clinical condition and the onset of peritonitis, the patient underwent an emergency exploratory laparotomy (Figure 4).

At surgical exploration, a defect of size 6 x 5 cm was identified just behind xiphisternum through which part of the stomach, transverse colon, and omentum had herniated into mediastinum. After confirming viability by inspection, the contents were reduced back into abdomen. The rest of the gut, intra-abdominal structures, and organs were found to be normal. The diaphragmatic rent was repaired and plication of diaphragm done with non-absorbable suture. The post-operative course of the patient was uneventful, and the patient was discharged on post-operative Day 5. He has had complete resolution of symptoms and has had no recurrence. An upper gastrointestinal endoscopy done after three weeks was normal.

## Discussion

Morgagni’s hernia is the rarest of the congenital diaphragmatic defects with a reported frequency of 1% to 5.1% [3]. It has been hypothesised they occur due to failure of the pleuroperitoneal folds to close or due to genetic or environmental triggers that disrupt the differentiation of mesenchymal cells during the formation of the diaphragm [4-5]. Morgagni hernias are more commonly seen on the right side despite protection from the liver. It has been suggested that this happens because the presence of extensive pericardial attachments on the left provides extra support [2]. It occurs more commonly in females and is associated with other anomalies such as ventricular septal defect, dextrocardia, anomalous pulmonary venous return, Down’s syndrome, Turner’s syndrome, Noonan’s syndrome, Prader Willi syndrome, tetralogy of Fallot, scoliosis, and omphalocele [2-3]. The contents of the hernia are usually the omentum, liver, colon, or the small bowel either occurring individually or in combination with one of the other contents. Most patients are asymptomatic, although vague epigastric discomfort may be the only symptom in many cases [6]. Despite being a congenital condition, many cases present only later in life. It is believed this is due to the presence of a sac which limits herniation. A preceding event like trauma or raised intra-abdominal pressure then causes rupture of the sac leading to herniation and subsequent appearance of symptoms. Another hypothesis is that herniation may occur early, but symptoms develop when the bowel is compromised [3].

On a chest radiograph, MH are frequently diagnosed incidentally as homogeneous masses in the right cardio phrenic angle. In case of involvement of a hollow viscus, an air fluid level may be noted in the thoracic cavity [7]. However, it must be noted that plain radiography may be normal especially in intermittent herniation [6]. CT scan is the investigation of choice for diagnosis of Morgagni’s hernia. The diagnosis is established by the presence of a retrosternal mass of fat density representing herniated omentum or by a combination of omentum and a hollow viscus organ [3].

Once established, treatment is dependent on the setting. Unless evidence of bowel compromise exits, surgery can be deferred until apt imaging is available [3, 8]. There are various different approaches to surgery. Both transthoracic and transabdominal approaches have been described. There are many reports of laparoscopic repairs of Morgagni’s hernias reported in the literature as well. For a smaller defect, primary closure is usually done, but in the case of larger defects, a mesh may be necessary [1, 3].

## Conclusions

Our patient is one of a handful of individuals reported to have presented with this congenital disorder at such an advanced age. This case illustrates the need to act quickly when a potential diagnosis of a Morgagni’s hernia is made. A missed diagnosis can lead to complications such as haemorrhage, obstruction, or strangulation which need early surgical intervention. Although the patient can be managed non-operatively, the onset of peritonitis is an indication for urgent surgical exploration.

## References

[1] Pfannschmidt J, Hoffmann H, Dienemann H (2004). Morgagni hernia in adults: results in 7 patients. Scand J of Surg.

[2] Horton JD, Hofmann LJ, Hetz SP (2008). Presentation and management of Morgagni hernias in adults: a review of 298 cases. Surg Endosc.

[3] Nasr A, Fecteau A (2009). Foramen of Morgagni hernia: presentation and treatment.. Thorac Surg Clin.

[4] Bielinska M, Jay PY, Erlich JM (2007). Molecular genetics of congenital diaphragmatic defects. Ann Med.

[5] Slavotinek AM (2005). The genetics of congenital diaphragmatic hernia. Semin Perinatol.

[6] Eren S, Gümüş H, Okur A (2003). A rare cause of intestinal obstruction in the adult: Morgagni's hernia. Hernia.

[7] Colakoğlu O, Haciyanli M, Soytürk M, Colakoğlu G, Simşek I (2005). Morgagni hernia in an adult: atypical presentation and diagnostic difficulties. Turk J Gastroenterol.

[8] Arora S, Haji A, Ng P (2008). Adult Morgagni hernia: the need for clinical awareness, early diagnosis and prompt surgical intervention. Ann R Coll Surg.

